# An Alternative Perfusion Approach for the Intensification of Virus-Like Particle Production in HEK293 Cultures

**DOI:** 10.3389/fbioe.2020.00617

**Published:** 2020-06-19

**Authors:** Jesús Lavado-García, Laura Cervera, Francesc Gòdia

**Affiliations:** Grup d'Enginyeria Cellular i Bioprocés, Escola d'Enginyeria, Universitat Autònoma de Barcelona, Barcelona, Spain

**Keywords:** bioreactor, perfusion, ATF, design of experiments, VLP, HFM

## Abstract

Virus-like particles (VLPs) have gained interest over the last years as recombinant vaccine formats, as they generate a strong immune response and present storage and distribution advantages compared to conventional vaccines. Therefore, VLPs are being regarded as potential vaccine candidates for several diseases. One requirement for their further clinical testing is the development of scalable processes and production platforms for cell-based viral particles. In this work, the extended gene expression (EGE) method, which consists in consecutive media replacements combined with cell retransfections, was successfully optimized and transferred to a bioreactor operating in perfusion. A process optimization using design of experiments (DoE) was carried out to obtain optimal values for the time of retransfection, the cell specific perfusion rate (CSPR) and transfected DNA concentration, improving 86.7% the previously reported EGE protocol in HEK293. Moreover, it was successfully implemented at 1.5L bioreactor using an ATF as cell retention system achieving concentrations of 6.8·10^10^ VLP/mL. VLP interaction with the ATF hollow fibers was studied via confocal microscopy, field emission scanning electron microscopy, and nanoparticle tracking analysis to design a bioprocess capable of separating unassembled Gag monomers and concentrate VLPs in one step.

## Introduction

Since the human immunodeficiency virus 1 (HIV-1) was first reported in 1959, 74.9 million people have been infected (Whiteside and Wilson, 2018). Due to the high impact and importance of this disease, many efforts have been devoted to understand and characterize the mechanisms taking place in HIV infection and replication (von Schwedler et al., 2003; Pincetic and Leis, 2009; Meng and Lever, 2013; Patters and Kumar, 2018). For replication and virion production, HIV-1 Gag polyprotein play a key role (Bell and Lever, 2013). Upon expression of this polyprotein, it self-assembles forming the virion protein core which can be later packed with the genetic material and bud off the host cell (von Schwedler et al., 2003; Jolly and Sattentau, 2007; Meng and Lever, 2013). This characteristic can be exploited to obtain protein core structures comprising Gag monomers and lacking genetic material. The use of Gag to generate virus-like particles (VLPs) has become a promising option to develop alternative platforms for recombinant vaccines being able to target several diseases (Lua et al., 2014; Cervera et al., 2019). Having multimeric structures such as Gag-VLPs helps induce a stronger immune response (Meador, 2017) and since they are produced by a budding process, the lipid envelope can be further used for particle pseudotyping (Kueng et al., 2007, 2011; Sharma et al., 2017). The addition of conjugated antigens can be used to target different diseases, such as influenza (Venereo-Sanchez et al., 2017). The use of mammalian cell cultures to express HIV-Gag in order to produce VLPs offers the advantage of providing the necessary cellular machinery to produce enveloped particles via membrane budding and performing the correct post-translational modification (Bandaranayake and Almo, 2014). One methodology to express heterologous proteins like Gag, is transient gene expression (TGE) (Gutiérrez-Granados et al., 2018a). It consists in the introduction of a plasmid carrying the gene encoding the protein of interest that will remain episomal in the nucleus, while it is being expressed. This expression method allows production of relatively high titers of protein in a short period of time, reduces time costs related to the generation of a stable cell line and offers high versatility when developing a production platform regardless of the final product, as only the plasmid of interest needs to be changed for a new product. Nevertheless, this method is limited in its timespan, since the plasmid is not integrated in the cell genome and upon consecutive rounds of cell division it is eventually lost. To overcome this issue and envisioning a future step toward industrialization, a protocol named Extended Gene Expression (EGE) was designed allowing to advance from a typical harvest at 72 h post transfection (hpt) to a continuous harvest up to 240 hpt (Cervera et al., 2015). The EGE protocol increased protein production up to 12-fold and consisted of a combination of media replacements every 48 h and two retransfections at 48 and 96 hpt. The fact of having a constant rate of media replacement makes the EGE protocol suitable for continuous biomanufacturing using cell retention devices. Continuous production allows the constant renewal of nutrients and depletion of cell waste, toxic by-products or metabolites that might interfere with the culture homeostasis (Butler, 2005). As a result, higher volumetric productivity can be reached compared to batch and fed-batch culture. Indeed, the reduction of bioreactor size and facilities infrastructure, with a reduction in production costs, is facilitating the adoption of continuous processes in the biopharmaceutical industry over the last few years (Gutiérrez-Granados et al., 2018b). One of the main advantages of continuous bioprocessing is that this mode of operation allows the support of high cell density cultures, dramatically increasing volumetric productivity as the product is being constantly harvested and operation times extended. The fact of achieving higher cell densities in shorter times was one of the issues encountered in the first attempt of transferring the EGE protocol to a bioreactor operated in continuous mode without further optimization (Fuenmayor et al., 2019). Indeed, cells need to be maintained around 2·10^6^ cells/mL for an efficient transfection. Higher cell densities are more difficult to transfect due to the widely described cell density effect (CDE) (Bernal et al., 2009; Le Ru et al., 2010; Petiot et al., 2015). Transfection efficiency and productivity decrease when performed at higher cell densities. The energetic demand is solved with the constant addition of fresh media but the CDE comprises molecular causes that cannot be solved only by perfusion (Petiot et al., 2015). In this work, we propose overcoming this problem by changing the approach of perfusion using cell retention devices, like alternating tangential flow (ATF), not to achieve high cell densities, but to ensure the constant media replacement in order to maintain the cell culture in a re-transfectable state for a longer period of time in the bioreactor and therefore, prolong production. This perfusion approach is not intended to operate at steady state and cell bleeding is not necessary, as continuous perfusion is used to constantly renew media while cells are retransfected to keep a high proportion of cells producing VLPs, up to a certain limited process span. To achieve a scalable optimization of the EGE protocol, key parameters important to improve production yields were considered. The time of retransfection, the cell specific perfusion rate (CSPR), and the DNA concentration added in each transfection were the three variables optimized using a Design of Experiments (DoE) method. VLP production was assessed and a new approach to develop the EGE protocol at bioreactor scale using ATF is suggested. This intensified and optimized procedure can reduce costs and increase VLP production for a future potential implementation at industrial scale, offering a production platform for novel VLP-based vaccines.

## Experimental Section

### HEK293 Mammalian Cell Line, Culture Conditions

The cell line used in this work is a serum-free suspension-adapted HEK 293 cell line (HEK293SF-3F6) kindly provided by Dr. Amine Kamen from McGill University (Montreal, Canada). Cells were cultured in disposable polycarbonate 125 mL flasks with vent cap (Corning®) at 37°C, 5% of CO_2_ and 85% RH at 130 rpm in a LT-X Kuhner shaker (LT-X Kuhner, Birsfelden, Switzerland). Cell culture media was HyCell™ TransFx-H media from HyClone™ (GE Healthcare, Chicago, IL, USA) supplemented with 4 mM GlutaMAX™ (Gibco, Life Technologies, ThermoFisher, San Jose, CA, USA) and 0.1% Pluronic™ F-68 Non-ionic Surfactant (Gibco, Life Technologies, ThermoFisher, San Jose, CA, USA).

Cell concentration and viability were determined using the NucleoCounter®NC-3000 automatic cell counter (Chemometec, Allerod, Denmark) according to manufacturer's instructions.

For the pseudoperfusion experiments, the total culture volume was 20 mL and media replacement (MR) was carried out centrifuging the culture at 300xg for 5 min every 12 h ensuring that the proportional volume of media was replaced depending on the condition. To maintain a MR rate of 2 reactor volume per day (RV/day), 20 mL were replaced every 12 h. For a rate of 1 RV/day, 10 mL were replaced every 12 h and for a rate of 0.5 RV/day, 5 mL were replaced every 12 h.

### Bioreactor Culture Conditions and Setup Description

A BioStat B Plus bioreactor (Sartorius Stedim Biotech, Goettingen, Germany) equipped with a 3-blade segment dual impeller with UP-DP configuration (Buffo et al., 2016) was used for HEK293 cell cultivation. The agitation was set a 200 rpm. Temperature was set a 37°C. pH was set at 7.1 and controlled with CO_2_ and NaHCO_3_ (7.5%w/v). Dissolved oxygen was controlled at 40% of air saturation by supplementing air by sparger at a constant flow of 0.1L/min and additional pure oxygen when needed. HEK293 growing exponentially in disposable polycarbonate 1 L shake flasks (Corning®) were used to seed the bioreactor at 0.5·10^6^ cells/mL in 1.5 L. Samples were taken every 24 h for cell counting and viability determination. After transfection, assessment of the percentage of GFP positive cells and VLP quantification was also performed every 24 h. Perfusion was achieved using an alternating tangential flow (ATF) cell retention device (Repligen, Waltham, MA, USA) with 0.5 and 0.2 μm pore size and 0.13 m^2^ of filtration area hollow fiber modules (Repligen, Waltham, MA, USA) and a ATF flow rate of 0.6 L/min. When performing transfection, perfusion was stopped to incubate the cells with the DNA/PEI solution and reestablished 2 h after transfection (Cervera et al., 2017). To carry out media replacement, the filtration rate was set at 0.26 mL/min at the beginning of the process using a MasterFlex L/S peristaltic pump (MasterFlex Group, Gelsenkirchen, Germany) and was modified every day depending on the viable cell density to maintain a cell specific perfusion rate (CSPR) of 30 pL/cell/day. The bioreactor and the ATF system were placed over a scale (MSE36200S-000-D0 Cubis, Sartorius, Goettingen, Germany) to control the mass displacement created due to the filtration of spent media. The scale was connected to a Scilog peristaltic pump (Scilog tandem 1081, Parker, Oxnard, CA, USA) which controlled the addition of fresh media upon detection of mass displacement to maintain constant volume in the bioreactor.

### Transient Transfection

The initial transfection (t_1_) was carried out at a cell density of 2·10^6^ cells/mL using a final DNA concentration of 1 μg/mL. PEI/DNA complexes were formed by adding PEI to plasmid DNA diluted in fresh culture media (10% of the total culture volume to be transfected). Transfection reagent PEIpro® (Polyplus-transfection, Illkirch-Graffenstaden, France) was used.

The plasmid used contained the gene coding for HIV-Gag protein fused to eGFP (Gag::eGFP). Briefly, the corresponding DNA was diluted with supplemented HyCell™ media and vortexed for 10 s. Then PEI was added in 1:2 (w/w) DNA:PEI ratio and vortexed three times, then the mixture was incubated for 15 min at room temperature and then added to the cell culture.

In the different series of perfusion experiments performed, the first and second retransfections (t_2_ and t_3_) were carried out following the previous protocol and incorporating some variations depending on each of the optimization experiments. The specific conditions used in each case are detailed in the *Results and Discussion* section.

### Flow Cytometry

Samples were taken every 24 h after transfection and cells were fixed using formaldehyde 2% for 10 min, centrifuged and then resuspended in PBS for FACS analysis. The percentage of GFP positive cells was assessed using a BD FACS Canto flow cytometer (BD Biosciences, San Jose, CA, USA). Laser 488 was used for GFP measurement. The results were analyzed with FACS DIVA software (BD Biosciences, San Jose, CA, USA).

### HIV-1 Gag VLP Quantification by Fluorimetry

The concentration of HIV-1 Gag VLPs was assessed by fluorimetry using a developed and validated quantification assay (Gutiérrez-Granados et al., 2013). VLP containing supernatants were recovered by cell culture centrifugation at 1000×g for 5 min. Relative fluorescence unit values (RFU) were calculated by subtracting fluorescence unit (FU) values of non-transfected negative control samples.

### HIV-1 Gag VLP Quantification by Nanoparticle Tracking Analysis

Nanoparticle Tracking Analysis (NTA) was also used to quantify fluorescent particles. NTA measurements were performed with a NanoSight® LM20 device (NanoSight Ltd., Amesbury, UK) equipped with a blue laser module (488 nm) to quantify HIV-1 Gag::eGFP VLPs and neutral density filter for total particle by light scattering. Data was analyzed with NanoSight® NTA 3.1 software. Briefly, samples were injected, and independent analyses were carried out. Three video recordings of 60 s duration were taken for each sample. Capture settings were recorded with an sCMOS camera (camera level of 8 for Gag::eGFP VLP samples, and 11 for controls, viscosity: 0.9 cP) and analyzed with a detection threshold of 4.

### Field Emission Scanning Electron Microscopy (FESEM) Visualization

Morphometry of the hollow fiber inner layer and fluorescence detection at nanoscale were determined by Field Emission Scanning Electron Microscopy (FESEM). The analyzed samples were 0.5 μm pore size hollow fiber samples exposed to non-transfected HEK293 as a control, 0.5 and 0.2 μm pore size hollow fibers exposed to VLP-producing HEK293 cell cultures. Hollow fibers were longitudinally cut in pieces of ~2–3 mm^2^ and deposited in carboncoated gold grids (200 mesh) during 1 min, air dried and observed in a FESEM Zeiss Merlin (Zeiss, Jena, Germany) operating at 1.5 kV and 3.4 mm of working distance. Samples were then randomly checked with an in-lens secondary electron detector for morphology and with a Back-scattered Electron (BSE) detector for fluorescence detection. Representative images were obtained at a wide range of high magnifications (from 200,000x to 500,000x).

### Confocal Microscopy Visualization

The visualization of the hollow fiber modules was performed using a FluoView®FV1000 confocal microscope (Olympus, Tokyo, Japan) at sampling speed of 2 μm/pixel, excitation at 488 nm and detection at 500–600 nm. Step size was 2 μm/slice using XYZ scan mode. Transversal and longitudinal cuts of the hollow fiber were made from samples of 0.5 μm pore size exposed to non-transfected HEK293 as a control and 0.5 and 0.2 μm pore size hollow fibers exposed to VLP-producing HEK293 cell cultures. For the longitudinal cuts, objective lens UPLAPO 20x, NA: 0.75 and optical zoom x3 was used. For the transversal cuts, objective lens UPLAPO 10x, NA: 0.40 and optical zoom 1x was used.

### Optimization of Retransfection Parameters Using Design of Experiments

Retransfection parameters were optimized in order to maximize VLP specific productivity. The three variables chosen for optimization were the time of retransfection, the cell specific perfusion rate (CSPR) and the DNA concentration added in each retransfection. The adjusted VLP specific productivity (*P*_*sp*_: VLP·10^6^ cells^−1^·days^−1^) for the DoE response was calculated taking into account the volume of media replacement and the cell culture viability in each day along the culture using the equation showed below (Equation 1):

(1) $$\begin{array}{r} P_{sp}=\frac{\sum_{i=1}^{d}\left(\frac{C_{VLP}}{C_{x}}\;K_{MR_{i}}v_{i}\right)}{d} \end{array}$$

(2) $$\begin{array}{r} K_{MR}=\;\{\begin{array}{c} \frac{MR}{RV}<1\;1-\frac{MR}{RV}\\ \frac{MR}{RV}>1\;\frac{MR}{RV} \end{array} \end{array}$$

where *C*_*VLP*_ is the VLP concentration (VLP/mL), *C*_*x*_ is the concentration of viable transfected cells (10^6^ cells/mL), *v*_*i*_ is the cell viability (%), *d* is the number of culture days after the first transfection and *K*_*MR*_ is the media replacement coefficient, proportional to the total volume replaced each day. *K*_*MR*_ is calculated as shown in Equation 2, where *MR* is the replaced media and *RV* is the reactor volume. Thus, estimating the final volume at the time extracellular fluorescence was measured. A Box-Behnken design was selected to define the optimal value for each variable. The three variables were screened at three levels: a low level coded as −1, an intermediate level coded as 0, and a high level coded as +1 as indicated in Table 1. Box-Behnken experimental results were fitted to a second order polynomial equation described below (Equation 3) by non-linear regression analysis: where *Y* is the response in VLP·Mcell^−1^·days^−1^, β_0_ is the offset term, β_*i*_ is the linear coefficient, β_*ii*_ is the quadratic coefficient, β_*ij*_ is the interaction coefficient, and *X*_*i*_ and *X*_*j*_ are the independent variables. This equation was used to predict the optimal values of the independent variables

(3) $$\begin{array}{r} Y=\beta_{0}+\sum \beta_{i}X_{i}+\sum \beta_{ii}\overset{2}{\underset{i}{X}}+\sum \beta_{ij}X_{i}X_{j} \end{array}$$

using R software (RStudio, Inc., Boston, MA, USA). The quality of the fit of the model equation is expressed by the coefficient *R*^2^ and *p-values* obtained by regression analysis. Additionally, a lack of fit test was performed to compare the experimental error to the prediction error. The overall significance of the model was determined by analysis of variance (ANOVA) F test, whereas the significance of each coefficient was determined by the corresponding t test.

**Table 1:** Box-Behnken design, results and ANOVA analyses for optimization of the extended gene expression (EGE) protocol for VLP production.

		−1	0	1
Retransfection time (hpt)		24	48	72
CSPR (pL/cell/day)		30	515	1000
DNA (μg/mL)^a^		0.5	1.25	2
**Experimental Run**	**Retransfection time**	**CSPR**	**DNA/mL**	**P**_**sp**_
1	1	0	−1	1.83E+09
2	1	0	−1	2.20E+09
3	−1	0	−1	2.68E+09
4	−1	0	−1	3.29E+09
5	1	0	1	1.85E+09
6	1	0	1	1.92E+09
7	0	−1	−1	5.06E+09
8	0	−1	−1	4.33E+09
9	0	1	−1	2.32E+09
10	0	1	−1	2.36E+09
11	0	1	1	2.10E+09
12	0	1	1	2.47E+09
13	−1	1	0	4.64E+09
14	−1	1	0	2.91E+09
15	1	−1	0	5.21E+09
16	1	−1	0	5.56E+09
17	0	0	0	2.07E+09
18	0	0	0	1.92E+09
19	0	0	0	1.72E+09
20	0	0	0	1.89E+09
21	0	−1	1	5.19E+09
22	0	−1	1	4.10E+09
23	1	1	0	2.25E+09
24	1	1	0	1.94E+09
25	−1	0	1	2.40E+09
26	−1	0	1	4.07E+09
27	−1	−1	0	5.65E+09
28	−1	−1	0	5.19E+09
29	0	0	0	2.12E+09
30	0	0	0	2.38E+09
**Model**	**Multiple** ***R***^**2**^	**p value** ^b^	**Lack of fit** ^c^	
	0.9126	1.06E-08	0.9316	
**Parameters**	**Co-efficient**		***t***	**P** ^b^
Constant	2.02E+09		10.225	<0.0001
[Time]	−5.04E+08		−4.1761	0.0005
[CSPR]	−1.21E+09		−9.9874	<0.0001
[DNA]	1.88E+06		0.0155	0.988
[Time]·[CSPR]	−4.11E+08		−2.4077	0.026
[Time]·[DNA]	−9.50E+07		−0.5562	0.584
[CSPR] · [DNA]	−1.25E+06		−0.0073	0.994
[Time]^2^	5.95E+08		3.3492	0.003
[CSPR]^2^	1.56E+09		8.7562	<0.0001
[DNA]^2^	−8.21E+07		−0.4617	0.649
**Optimal values**				
	Time of Retransfection	CSPR	DNA	P_sp_
	−1	−1	0.5977154	5.49E+09
	**At 24hpt**	**30 pL/cell/day**	**1.7 μg/ml**	

a *DNA/PEI ratio was always maintained at [1:2].*

b *p values under 0.05 are considered statistically significant with 95% confidence, and p values under 0.1 are considered statistically significant with 90%*.

c *p values associated to lack of fit test above 0.05 mean that the hypothesis arguing that the model is suitable cannot be rejected*.

*hpt: hours post transfection*.

## Results and Discussion

### Study of the Cell Growth Upon Different Media Replacement Rates

**Figure 1:**
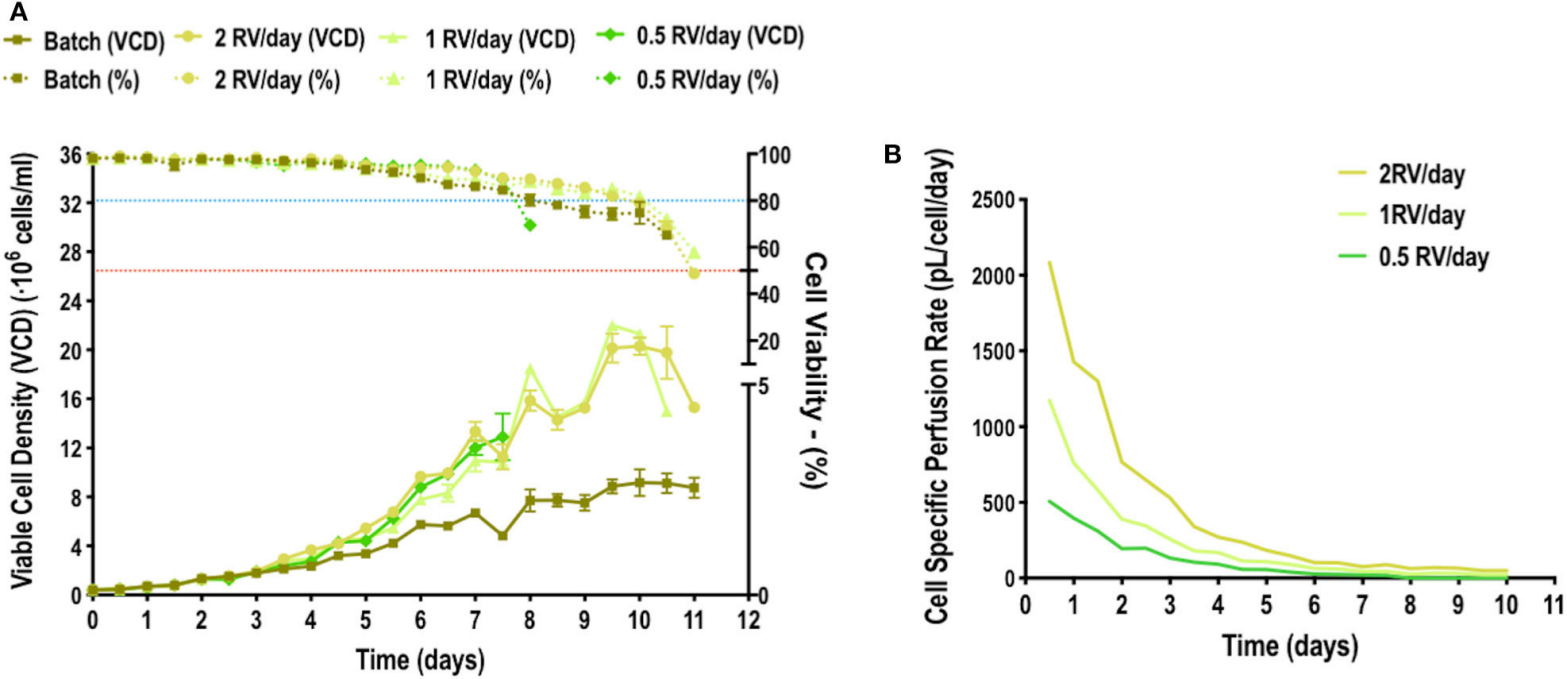
Pseudoperfusion growth curves **(A)** HEK293 cell culture growth curves in shake flasks where media replacement was performed at a rate of 0.5, 1, or 2 reactor volume per day (RV/day) and a batch condition where no media replacement was carried out. Blue and red dotted lines represent 80 and 50% of cell viability, respectively. Experiment performed *n* = 3. **(B)** Representation of the cell specific perfusion rate (CSPR) from cell cultures with different rate of media replacement.

Monitoring cell growth under three different media replacement rates showed that maximum cell concentration, reaching 22·10^6^ cells/mL, was achieved in the shake flask replacing media at 1RV/day and no difference was observed at 2RV/day (Figure 1A). This suggests that 1RV/day is enough to meet the nutrient demand of the culture and further cell growth could be hindered by other factors such as oxygen diffusion in the flask. Cell growth monitoring was ceased when viability reached 50% or below and no more data from that condition was acquired. The observed fluctuations are due to the process of centrifugation and resuspension, which added an intrinsic error to the monitoring process. However, this fluctuation does not affect the following CSPR calculation. When the CSPR is calculated for every time point in each condition (Figure 1B) it can be observed that it tends to converge to a range going from 30 to 60 pL/cell/day range, suggesting that maintaining the media replacement rate within this interval is enough to maintain cell growth.

### Optimization of Retransfection by Design of Experiment (DoE) Method

**Figure 2:**
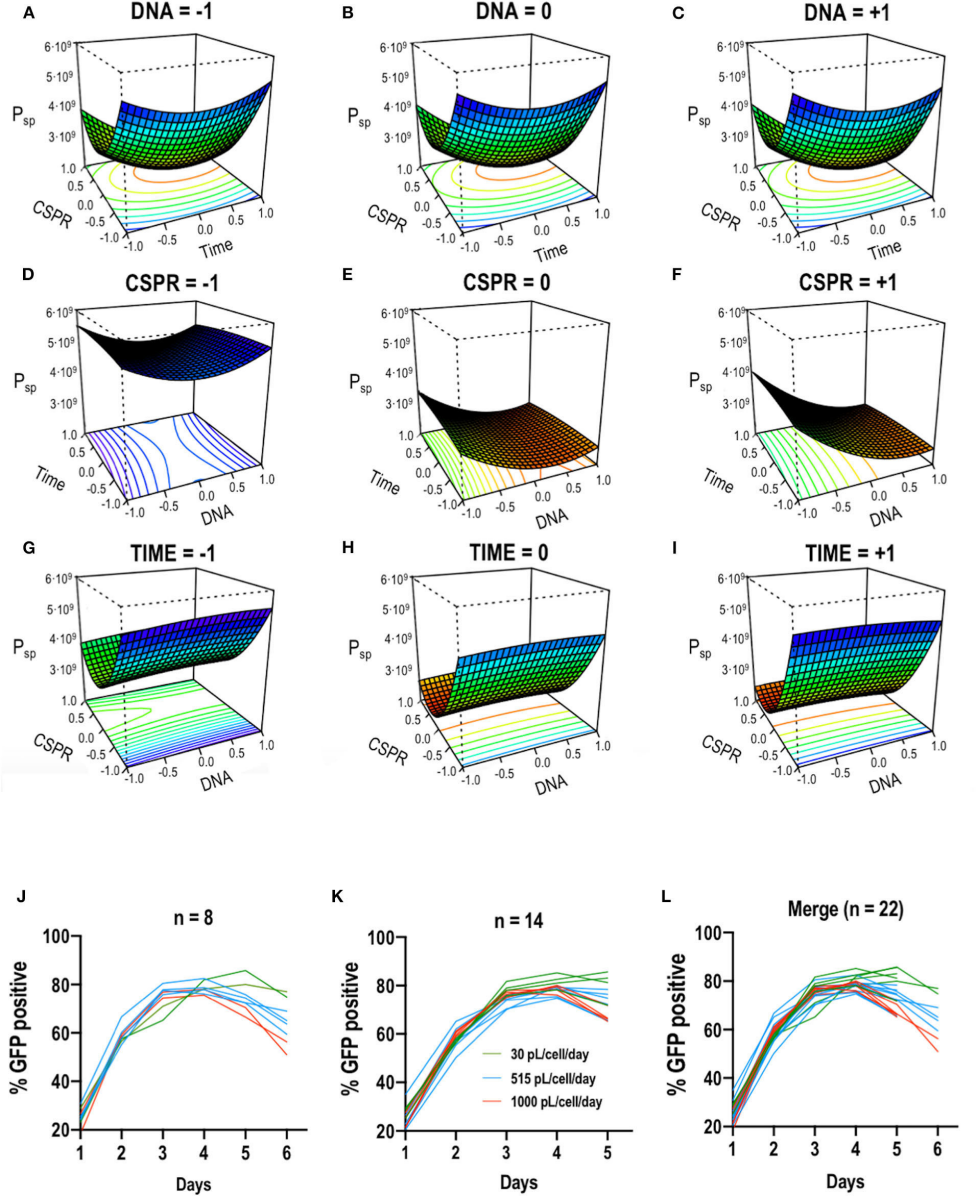
Response surface graphs based on Box-Behnken experimental results. Maximum VLP specific production in cell culture supernatants as a function of **(A–C)** CSPR vs. Time; **(D–F)** time vs. DNA; and **(G–I)** CSPR vs. DNA. The graphs were constructed by depicting two variables at a time and maintaining the third one at a fixed level. +1, 0, and −1 correspond to 0.5, 1.25, and 2 mg/mL for the DNA concentration; 30, 515, and 1000 pL/cell/day for the CSPR and 24, 48, and 72 hpt for the time of retransfection. **(J–L)** represent the changes of the percentage of transfection measured in percentage of GFP positive cells along the studied time course for conditions reaching the sixth day of the process **(J)** and conditions reaching the fifth **(K)**. **(L)** Shows the merged plot.

In order to optimize the EGE protocol, the continuous variables *time of retransfection, CSPR* and the *DNA concentration* used to retransfect, were selected. These variables were studied by means of a design of experiments (DoE). A response surface methodology (RSM), specifically a Box-Behnken design, was selected as it allows to reduce number of experimental runs and yet obtain statistically relevant information (Montgomery, 2019). The parameters of the first transfection point had been already optimized in previous work and fixed in 2·10^6^ cells/mL and 1 μg/mL of DNA (Cervera et al., 2013; Fuenmayor et al., 2018). The variables of [DNA:PEI] ratio, nature of the complexation agent and time for the maturation of the DNA:PEI complexes were also individually determined in previous works (González-Domínguez et al., 2019a,b). The range of DNA was also selected based on this previous experience and most importantly, restrained by the PEI cytotoxicity. In addition to this, multiple variable optimization was selected for the presented study since VLP production wanted to be tested combined with CSPR and time of retransfection to take into account possible synergic effects and simulate the closest condition to the bioreactor. The ratio [DNA:PEI] was fixed to [1:2] and maintained for all the experiments following the standard procedure. Regarding the time of retransfection, the working range was set from 24 to 72 hpt, based on the EGE protocol where the first retransfection was performed at 48 hpt. As for the CSPR, the working range was set based on the previous study of the cell growth upon different media replacement rates. The upper limit was set at 1000 and the lower limit at 30 pL/cell/day. Since the DNA concentration used for retransfection in the original EGE protocol was 0.5 μg/mL, the lower limit was set at this value. The upper limit for the DNA was set in 2 μg/mL due to the cytotoxic effects of PEI (Cervera et al., 2013; Gutiérrez-Granados et al., 2016). Working ranges for each variable are presented in Table 1. A three-factor three-level Box-Behnken design was constructed using the selected ranges for each variable as the design space boundaries. A 15-experiment matrix was defined in which the central point was triplicated, and each experimental run was duplicated to assess the pure experimental error. This led to a 30-experiment matrix (Table 1). Each condition was retransfected at its corresponding time and harvested 72 h after. The response variable considered in this analysis was the adjusted VLP specific productivity (*P*_*sp*_) calculated as described in Equation 1. This equation is proposed to optimize specific productivity taking into account two aspects of VLP production. A media replacement coefficient (K_MR_) was added, reflecting how many times a reactor volume of media was replaced every 12 h, making it directly proportional to the amount of replaced media to favor the conditions where the VLP concentration in the supernatant was diluted due to a higher replacement rate. A term reflecting cell viability (*v*) was also added to penalize conditions with low viability which are incrementing extracellular relative fluorescent units (RFUs) due to the release of unassembled free monomer caused by cell death. This is one of the reasons why specific productivity was chosen over total volumetric productivity as the DoE response to be based on. Otherwise conditions reporting high fluorescence intensity, but low cell densities and viabilities had been favored. Data was fitted to a second-order model by non-linear regression analysis. The statistical significance of the non-linear regression was confirmed by ANOVA analysis, also showed in Table 1. Data from the model was used to construct response surface plots where the interaction between factors can be observed (Figure 2). Noticeably, lower CSPRs led to higher adjusted specific productivities (*P*_*sp*_) (Figures 2D–F). This condition allows to minimize media consumption while increasing productivity. Higher CSPRs led to the same cell densities at the moment of transfection, but the transfection percentage dropped faster in these conditions as the subpopulation of non-transfected cells grew faster than the transfected one, losing the plasmid more rapidly (Figures 2J–L). This can be explained as overall cell homeostasis is disrupted upon transfection, reducing cell growth and downregulating the cell cycle (Lavado-García et al., 2020). Regardless of the time of retransfection, decreasing CSPR led to an increase in the specific productivity. It should also be considered that, due to cytotoxicity of the complexation agent (PEI), an increase in the DNA concentration compromises viability. The model was used to predict an optimal combination of the three factors to maximize *P*_*sp*_, reaching a value of 5.5·10^9^ VLP·Mcell^−1^·days^−1^. The optimal set of parameters for the first retransfection point was to perform it at 24 hpt using 1.7 μg/mL of DNA and maintaining a CSPR of 30 pL/cell/day. In order to validate the model, a confirmation experiment (*n* = 4) was performed using the set of parameters determined by the optimal solution predicted from the model. A *P*_*sp*_ of (5.7 ± 0.8)·10^9^ VLP·Mcell^−1^·days^−1^ was obtained, confirming the model adequacy.

### Optimization of the Second Retransfection Parameters Shake Flasks Study

Following the EGE protocol and after optimizing the first retransfection, a similar approach was performed to optimize the second retransfection. This time, based on the results from the first optimization, a CSPR of 30 pL/cell/day was maintained for all the experiments. In order to estimate the optimal values for the retransfection time and DNA concentration, a central composite design (CCD) was used. The same working range as in the Box-Behnken design was maintained for the DNA and for the time of retransfection, the working range was set from 24 to 72 h post retransfection. A 10-experiment matrix was defined, and each experimental run was duplicated to assess the experimental error. In this case, the temporal proximity of the three transfections was so close that cell toxicity caused by PEI made unfeasible the completion of the experiment for the tested conditions. Therefore, no statistically significant model was obtained (data not shown). As this approach did not succeed to optimize the second retransfection, a further analysis of the culture behavior once transferred to the bioreactor was carried out as discussed further.

### Intensification of the Optimized Protocol in Bioreactor Using ATF

**Figure 3:**
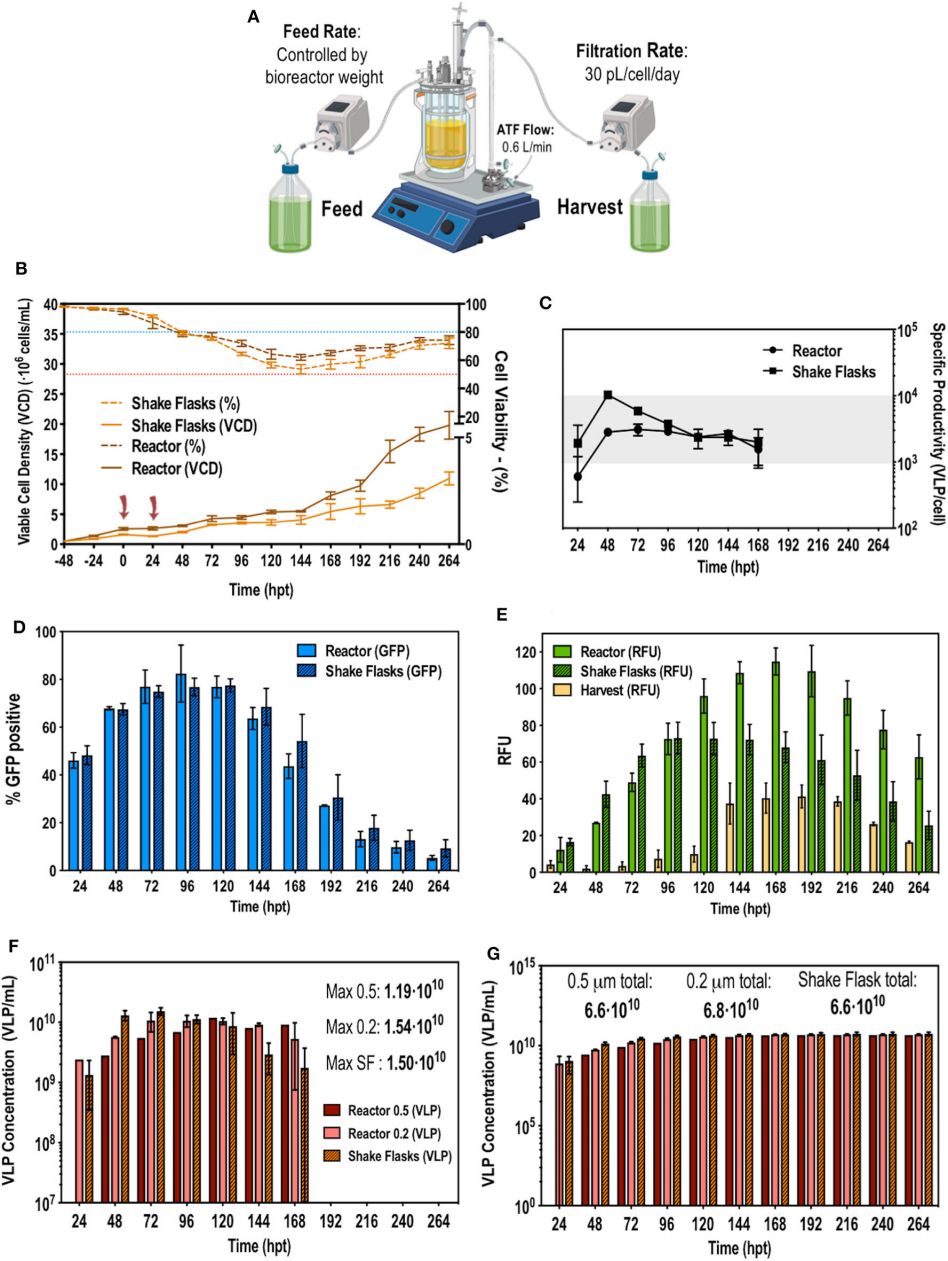
**(A)** Schematic representation of the implemented ATF perfusion system. **(B)** Viable cell density and cell viability graphs along the studied time course. The blue and red dotted line indicate 80 and 50% of cell viability, respectively. Cells were transfected at time point 0 and 24 h post-transfection (hpt) noted by red arrows. **(C)** VLP specific productivity rate along the studied time course. **(D)** Flow cytometry analysis of the transfection percentage (showed in GFP positive percentage of cells) along the time course. **(E)** Measurements of **f**luorescence in relative fluorescent units (RFUs) from the reactor, the harvest and the shake flasks. **(F)** Total VLP concentration produced per day. Two different pore size for the hollow fiber module were used in the ATF system: 0.5 and 0.2 μm denoted as 0.5 and 0.2, respectively. **(G)** Total cumulative VLP concentration. Experiments performed *n* = 3.

The ATF configuration previously described in the Methods section and illustrated in Figure 3A was implemented to apply the optimal solution obtained using the Box-Behnken design to the stirred tank bioreactor (STR) culture operating in perfusion mode. Transfecting at 2·10^6^ cells/mL at 0 h and then retransfecting at 24 hpt reduced cell growth, making the viable cell density (VCD) to remain stable at 3–4·10^6^ cells/mL up to 144 hpt (Figure 3B). Up to this point viability steadily dropped from 98 to 50%, following the same trend in both shake flasks (SF) and in the STR. From 144 hpt viability started to restore and cell growth recovered exponential rate until 264 hpt when the culture was stopped. In this phase of the culture, the STR reached 22–23·10^6^ cells/mL compared to the 10·10^6^ cells/mL observed in SF. Although the same CSPR was used for both systems, in the STR, perfusion was automatically and continuously operated while in SF a pseudoperfusion was carried out, manually replacing the corresponding amount of media every day. Operating in continuous mode proved to be more efficient as nutrients were continuously replenished and waste removed. The percentage of transfection and the fluorescence intensity were monitored daily. There was a peak in transfection at 96–120 hpt reaching 90% of transfected cells (Figure 3D) and a peak of production at 168 hpt reaching 115–120 RFUs in the bioreactor (Figure 3E). RFU were converted to VLP concentration using a quantification method by fluorimetry previously described Gutiérrez-Granados et al. (2013). Taking into account the VLP concentration in the STR, the harvest and the corresponding CSPR, the total VLP production per day was calculated. As it can be observed in Figure 3F, the system ceased producing VLPs at 168 hpt, indicating the 13 days process can be reduced to 9 days subsequently reducing time, resources and costs. Cumulative VLP production (Figure 3G) achieved was 6.8·10^10^ VLPs/mL, improving in 86.7% or 7.54 fold the previous reported results (Cervera et al., 2015) using the original EGE protocol in shake flasks. Comparing the EGE protocol carried out in bioreactor prior to the developed optimization (Fuenmayor et al., 2019), the presented work achieved a reactor and media volumetric productivity of 7.1·10^12^ and 2.7·10^12^ VLP·L^−1^·day^−1^, respectively, improving 26.8% or 1.36 fold and 67.8% or 3.1 fold, respectively, the reported results. This improvement led to the achievement of an average VLP specific productivity of 3,000 VLP·cell^−1^·day^−1^ (Figure 3C). The same VLP concentration, specific productivity and trend in transfection efficiency were achieved in SF and STR, proving that the optimized EGE protocol was successfully transferable to the 1.5L bioreactor.

### Optimization of the Second Retransfection Parameters Bioreactor Study

**Figure 4:**
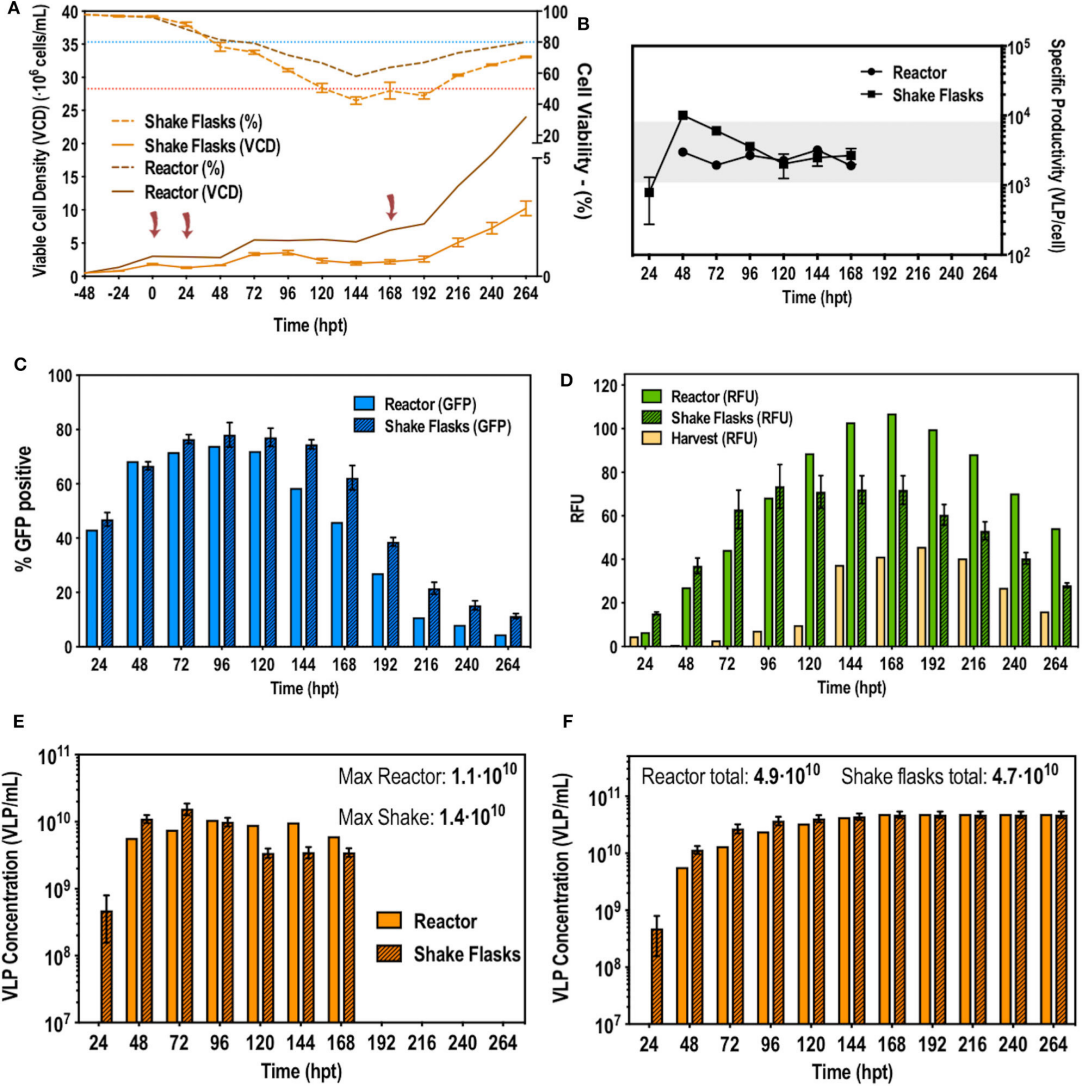
Second retransfection at 168 hpt in bioreactor. **(A)** Viable cell density and cell viability graphs along the studied time course. The blue and red dotted line indicate 80 and 50% of cell viability, respectively. Cells were transfected at time point 0, 24, and 168 h post-transfection (hpt) noted by red arrows. **(B)** VLP specific productivity rate along the studied time course. **(C)** Flow cytometry analysis of the transfection percentage (showed in GFP positive percentage of cells) along the time course. **(D)** Measurements of **f**luorescence in relative fluorescent units (RFUs) from the reactor, the harvest and the shake flasks. **(E)** Total VLP concentration produced per day **(F)** Total cumulative VLP concentration.

After the difficulties of the first attempt of optimizing a second retransfection at small scale using pseudoperfusion, a different approach was taken. Taking into account the trends in cell viability, a second retransfection following the standard protocol with 1 μg/mL of DNA, was carried out at 168 hpt. At this point, viability surpassed 60% and started recovering. Cells need to be growing to be efficiently transfected as they need to be dividing for exogenous DNA to enter the nucleus (Cervera et al., 2017). Therefore, at 168 hpt their recovering state would allow a new retransfection. In addition to this, at 168 hpt VLP production ceased. At this moment the VCD was 7–8·10^6^ cells/mL. Figure 4 illustrates the different studied process variables such as specific VLP production, percentage of transfection and total VLP production. Interestingly, this second retransfection had no effect. VCD and cell viability maintained the trend previously observed and no additional changes in transfection and production were observed. As a result, a second retransfection was discarded. This can be explained by the cell density effect (CDE), which shows the difficulty to transfect cell cultures at high cell densities (Petiot et al., 2015). Indeed, these results confirmed the CDE, which was originally reported to be related to energy depletion (Bereiter-Hahn et al., 1998; Bernal et al., 2009; Petiot et al., 2015). However, perfusion cultures are fed with a continuous addition of fresh media and removal of metabolic waste, preventing energy depletion. Encountering the CDE in continuous cultures has been already reported (Genzel et al., 2014; Fuenmayor et al., 2019) and different molecular and metabolic factors have been described to influence the CDE (Lavado-García et al., 2020). Therefore, using perfusion to achieve very high cell densities, as implemented when working with stable gene expression (SGE) cell-based platforms, is no longer useful for bioprocesses using TGE. Instead, the ATF perfusion system can be used to maintain a transfectable cell density and perform perfusion to maintain the constant media replacement in order to prolong production a defined period of time.

### VLP Retention by the ATF Hollow Fiber Module

**Figure 5:**
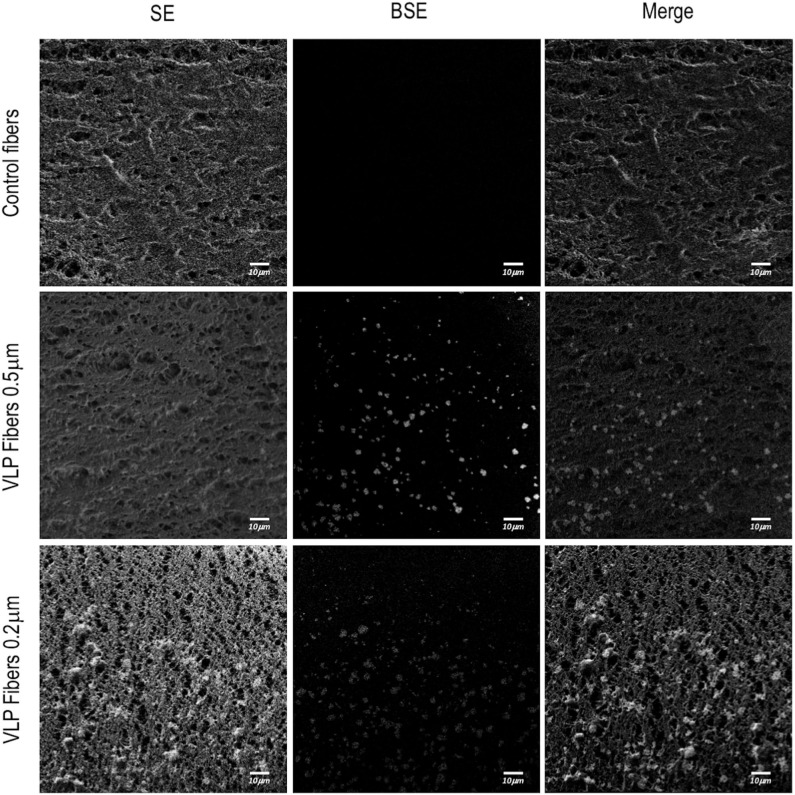
Visualization of a ATF hollow fiber inner side from a longitudinal cut using field emission scanning electron microscopy (FESEM). Rows represent the different studied conditions: control fibers used in non-producing-VLPs HEK293 cultures and fibers used in VLP-producing HEK293 cultures of 0.5 and 0.2 μm of pore diameter. Images were taken at 3.4 mm of working distance and 129,000x. The first column shows the morphology of the fibers using scanning of secondary electron (SE) detection. The second column shows back-scattered electron (BSE) detection for fluorescence detection from the same area. The third column shows the merge of the SEM image and the electron back scattering acquisition.

**Figure 6:**
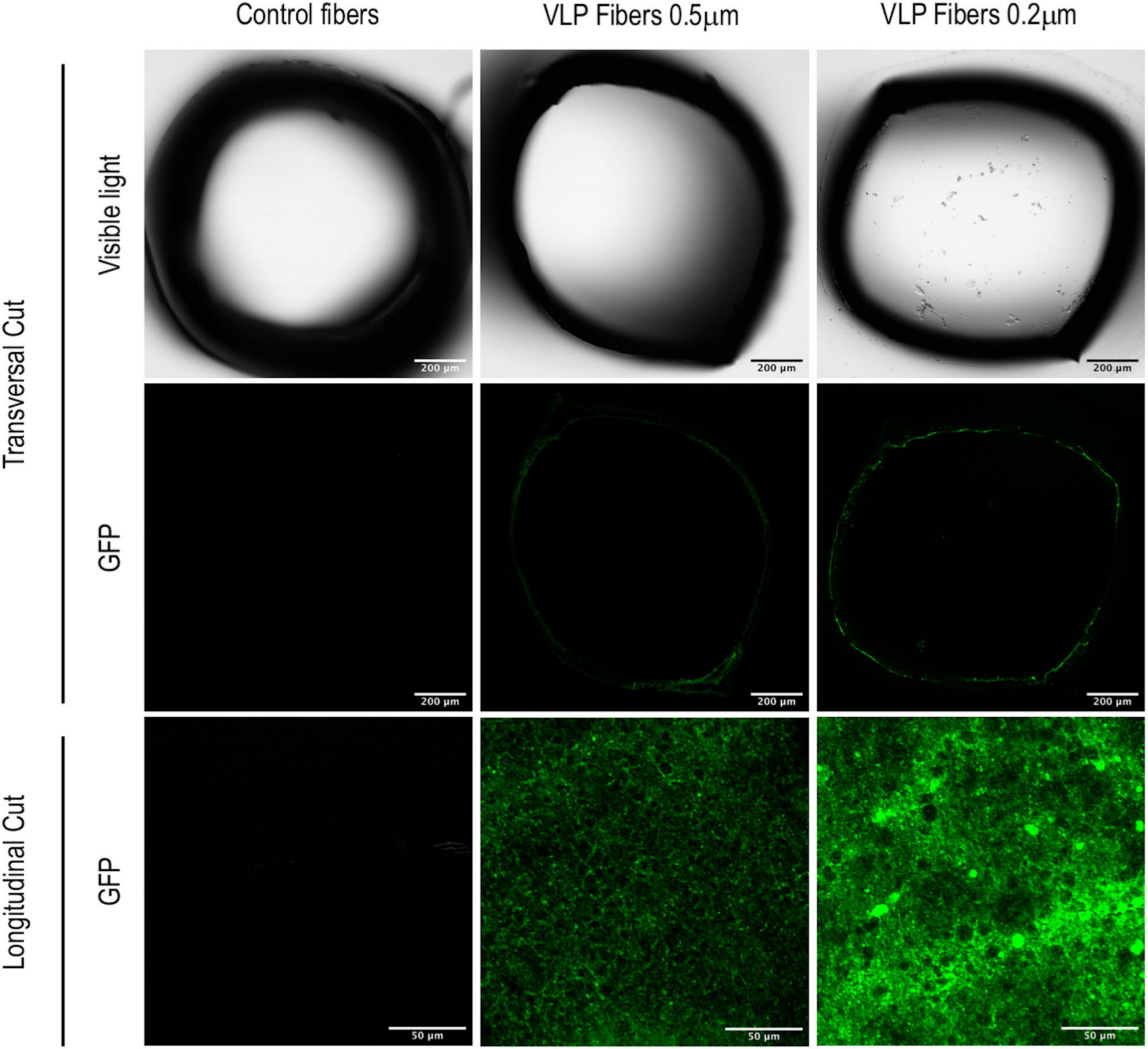
Visualization of ATF hollow fibers using confocal microscopy. Columns represent the different studied conditions: control fibers used in non-producing-VLPs HEK293 cultures and fibers used in VLP-producing HEK293 cultures of 0.5 and 0.2 μm of pore diameter. The first row shows transversal cuts of the fibers under visible light. The second row shows GFP fluorescence of the same transversal cut. The third row shows GFP fluorescence from a longitudinal cut of the luminal space.

For the ATF configuration, hollow fiber modules (HFM) with pore sizes of 0.5 and 0.2 μm were used in order to compare their VLP recovery efficiency. Regardless of the HFM, transfection efficiency and total VLP production remained constant (Figures 3F–G). However, despite having a diameter of 140 nm, VLPs were clearly retained inside the bioreactor. Therefore, a detailed study of VLP retention by the HFM was carried out. In order to characterize the interaction between VLPs and the HFM, confocal and field emission scanning electron microscopy (FESEM) studies were performed. In these analyses for characterization of VLP retention in the ATF system, a sample from fibers exposed to conditioned media free of VLPs from non-transfected HEK293 culture was used as a control. Longitudinal cuts of the lumen of the hollow fibers from the three conditions were visualized under FESEM to evaluate the morphology and presence of nanoparticles deposited on the fiber (Figure 5). Detection of secondary electrons (SE) is routinely used to evaluate size, size distribution and topography while detection of backscattered electrons (BSE) reveals differences in chemical composition and allows the detection of nanoparticles (Kowoll et al., 2017; Österreicher et al., 2018) and substrates with different composition deposited on the fiber surface. Contrast of BSE for imaging has been widely used for metal nanoparticles but it can also be used to detect fluorophores or molecules with electron excitation states whose electronic rearrangement after being exited under the electron beam, behave like a heavier atom. BSE originate from the elastic scattering of primary electrons upon interaction with the nucleus of the atoms in the sample. Therefore, atoms with heavier nuclei cause stronger scattering, providing a more intense signal for the BSE detector. Fluorescent molecules produce a different BSE signal compared to the non-fluorescent background (Vancová and Nebesárová, 2015; Seras-Franzoso et al., 2016; Garming et al., 2017; Fokkema et al., 2018). The SEM analyses of the different fiber samples revealed that there is a deposit of particles in the fibers exposed to VLPs. The morphology of polyethersulfone (PES) fibers showed no difference but the BSE analyses showed clusters of particles of different chemical composition and a higher BSE intensity compared to the inert PES. In order to confirm the retention and deposit of VLPs in both 0.5 and 0.2 HFM, transversal and longitudinal cuts were also visualized using confocal microscopy (Figure 6). The transversal cuts revealed that fluorescent deposits appeared in the luminal space of the fibers in the conditions exposed to VLPs. Interestingly, the longitudinal cuts analysis showed fluorescent deposits following the same clustering pattern previously detected using FESEM. Coherently, in both transversal and longitudinal cuts, the detected fluorescence intensity increase in the 0.2 μm HFM due to smaller pore and higher retention. Therefore, it is proved that VLPs are being retained in the HFM forming clusters of particle aggregates of ~2.5 and 5 μm of diameter in the 0.5 and 0.2 μm HFM, respectively, with consistent results via confocal microscopy and SEM. Nevertheless, the amount of deposited VLP was negligible as VLP production in 0.2 and 0.5 conditions were equivalent (Figure 3G). The use of HFM has been previously reported to lead to particle retention. Especially when manufacturing viral particles, as hollow fibers are used to concentrate VLPs (Negrete et al., 2014). Significant retention of viruses has been reported when using ATF as the retention device for production of viral particles. In the case of viruses like Influenza A which are about 80–100 nm (Genzel et al., 2014; Coronel et al., 2019), modified vaccinia Ankara (MVA) virus (Vázquez-Ramírez et al., 2018) or even the production of yellow fever and Zika virus, both about 50 nm, recoveries <1% of the virus titer in the harvest fraction have been reported (Nikolay et al., 2018). Therefore, VLP retention observed in this work agrees with the already described results of viral particle retention.

### Quantification of VLP Retention

**Figure 7:**
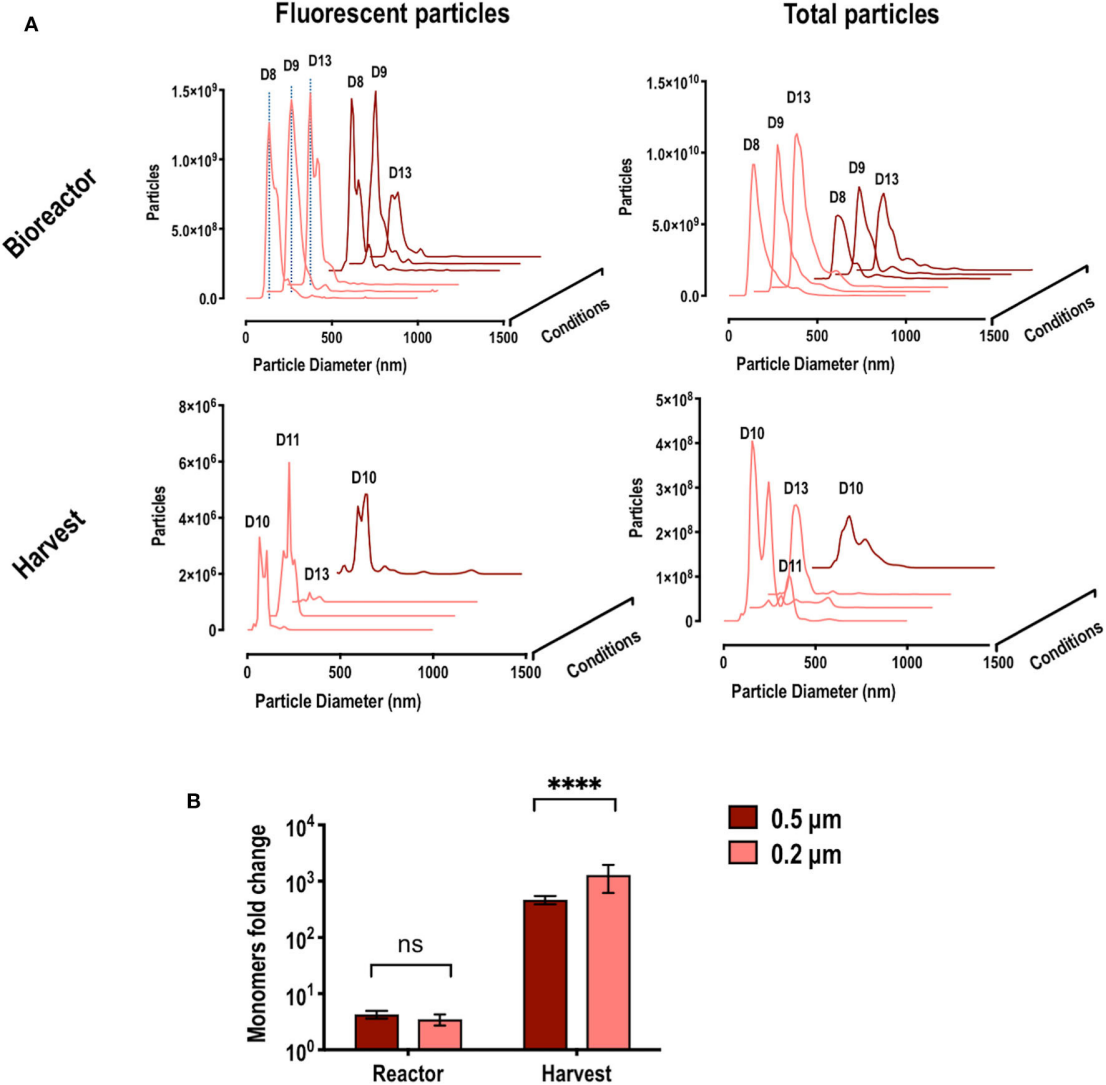
**(A)** Nano particle tracking analysis (NTA) from cultures where hollow fiber modules (HFM) with pore size of 0.5 and 0.2 μm were used. The left column shows the particle size distribution of the detected fluorescent particles (VLPs) along different days of the culture, in the reactor and harvest. The right column shows the particle size distribution of all particles detected (VLPs and extracellular vesicles) along different days in the reactor and harvest. Dotted blue lines indicate particle size of 140 nm. D labels indicate the time (day of the bioprocess) **(B)** Fold change ratio between the estimated particle concentration using fluorimetry and the detected actual particle concentration using NTA. This fold change suggests the presence of unassembled free Gag monomers. Significance calculated using two-way ANOVA, DF = 6 and n=3 in each condition.

NTA analyses of samples from the bioreactor and from the harvest fraction were performed at different days to evaluate VLP quality and percentage of fluorescent particles in the different fractions and to determine the percentage of retention (Figure 7A). The size distribution curve of the fluorescent particles in the bioreactor, regardless of the HFM used, described particles of 140 nm and was maintained along the whole bioprocess, confirming the stability of the particles. More VLPs were retained in the 0.2 compared to the 0.5 μm HFM along the process. The measurement of total particles revealed that, as expected, the 0.2 μm HMF was retaining a greater total amount of particles inside the bioreactor compared to the 0.5 μm HFM. However, 12–15% of fluorescent particles was observed in the bioreactor in both cases. Surprisingly, when measuring the fluorescent particles found in the harvest fractions, the concentration found was significantly lower than expected. The size distribution curve showed an heterogeneous population of particles, representing only the 0.08–0.1% of the total particles found in the harvest, in both HFM used. Calculations for both HFM revealed a 99% of VLP retention inside the bioreactor.

Comparing the value of VLP concentration obtained by the fluorimetry-based and NTA methods of quantification, ratios reflecting the presence of extracellular unassembled free fluorescent Gag monomers can be assessed. Samples with a high concentration of free monomer will provide a high fluorescence intensity signal while presenting a low particle concentration by NTA. Inside de bioreactor, the observed ratio VLP:monomer is around 1:4 while in the harvest fraction can reach up to 1:1762. These ratios are showed in Figure 7B. The free monomer ratio observed in the bioreactor did not depend on the HFM, as the difference found comparing 0.5 and 0.2 μm HFM was not significant. On the other hand, the ratio found in the harvest was around 10^3^ and significantly changed depending on the HFM used. The bigger pore of the 0.5 μm HFM facilitated the filtration of free monomer together with VLPs compared to the 0.2 μm HFM, reducing the ratio VLP/free monomer to 1:816 while in the 0.2 μm HFM the ratio significantly increased to 1:1762 due to the separation of VLP and free monomer via its filtration through the fiber. This can explain the low percentage of fluorescent particles found in the harvest and can be used for the direct separation of unassembled free Gag monomer, providing substantial bioprocessing advantages as discussed further.

### Selection of Conditions for a VLP Production Platform

Considering the results obtained in the previous sections, the conditions for the implementation of the intensified and optimized EGE method at bioreactor scale using an ATF system for perfusion could be defined. The use of 0.2 μm HFM provided the most advantages for the development of a VLP-based production platform. The retention of the product within the reactor allowed the one-step production and concentration of VLPs, achieving 12–15% VLPs, compared to the total of extracellular vesicles, increasing from the previously reported 1% (Fuenmayor et al., 2019). Thus, eliminating the problem of having the total amount of VLPs diluted in the harvest fraction, increasing media volumetric productivity 67.8% or 3.1 fold. The problem of having the product retained might concern VLP stability but particles were proved stable at 37°C throughout the process, overcoming the stability issue. This might be due to the nature of enveloped particles. Biological membrane-bound extracellular vesicles, like exosomes and microvesicles, are stable at physiological conditions such as temperature and pH (Madison and Okeoma, 2015; Ellwanger et al., 2017), equivalent to the bioreactor conditions. In addition to this, the 0.2 μm HFM allows the separation of unassembled free Gag monomer in the same step. Hence, improving the consecutive downstream processes not only by increasing the initial VLP concentration regarding the total number of particles, such as exosomes or microvesicles, but also regarding the quality, separating VLPs from free extracellular monomers. Therefore, overcoming challenges typically associated with VLP purification. However, the CDE prevented production from being extended in time, not being able to prolong gene expression further than 168 hpt, when cells ceased producing. However, product retention in the bioreactor is forcing to stop the process at a harvest time point and to be restarted, as in batch culture mode. In order to operate continuously and not being restricted to the batch process limitations, a continuous approach is proposed. Future work will be performed to demonstrate the validity of such an approach. When reaching the VLP harvest point at 168 hpt, a bleeding step might be added to harvest most of the culture and maintain the corresponding amount of cells to seed the bioreactor again at 0.5·10^6^ cells/mL with fresh media and to recover the initial state, restarting the optimized EGE transfection process.

This novel perfusion approach is different to the conventional use of perfusion for achieving high cell densities and maintaining steady state through purge or bleeding. Here, continuous perfusion is used to keep a constant media replacement while bleeding would be used to recover the product every 168 hpt and continue the process. Although the recovered culture should undergo a clarification process to separate the cell mass from the VLPs in the supernatant, this method would allow the acquirement of higher volumetric productivities while maintaining the constant media replacement. Thus, overcoming the batch and fed-batch limitations, the implementation of Sequential Perfusion recovery (SPR) is the proposed mode of operation. Conventional perfusion processes using ATF has been reported to successfully run for 50–80 days (Clincke et al., 2013; Zhang et al., 2015; Schwarz et al., 2020). Therefore, the ATF membrane integrity should not be compromised for several 168-h cycles, depending on the desired VLP titer.

## Conclusions

The implementation of the extended gene expression protocol has been achieved at bioreactor scale in perfusion mode circumventing limitations previously observed, such as a unfavorable effect on the purity of the product. In this work, an approach based on the design of experiments method was applied to optimize the retransfection time, the CSPR and the DNA concentration. The optimal solution for the variables of retransfection at 24 hpt, a CSPR of 30pL/cell/day and a DNA concentration of 1.7 μm g/mL were used to perform the process in a 1.5L stirred tank bioreactor. The same volumetric productivity, specific productivity and VLP concentration were achieved in the parallel shake flask culture and the bioreactor. The process was successfully operated at a 1.5L, reaching a concentration of 6.8·10^10^ VLP/mL and a total cumulative particle amount of 8.7·10^13^ VLPs in the bioreactor, improving the original EGE protocol 86.7% or 7.54 fold and the first bioreactor approach using acoustic filter 67.8% or 3.1 fold. A summary of the process variables is presented in Table 2. Hollow fiber modules of 0.5 and 0.2 μm of pore size were tested when implementing the ATF perfusion system. A 99% of VLP retention in the bioreactor was observed in both alternatives, confirmed by FESEM, confocal microscopy and NTA analyses. Bearing this in mind, 168 hpt proved to be the optimal VLP harvesting time from the bioreactor. Interestingly, the 0.2 μm HFM provided the advantage of separating unassembled Gag monomers from correctly assembled VLPs and concentrating them in the bioreactor, facilitating the subsequent downstream processing. The use of the ATF system also allowed the intensification of the process, increasing production while reducing spent media. Although there is no previous work in VLP production by TGE using an ATF system for perfusion, the proposed platform reduce spent media from 9-10 to 3.8L and therefore resources and costs compared to the only additional reported study regarding TGE at bioreactor scale (Hong et al., 2019). Due to product retention, a proposed procedure of controlled cell bleeding at 168 hpt to harvest and restart the culture at an initial cell density of 0.5·10^6^ is proposed under the name of Sequential Perfusion Recovery (SPR). The cycle could be repeated as many times as needed to achieve the desired titer. This strategy is an intensified and optimized VLP production platform that could encourage the potential further development VLP-based therapies, potentially applicable to several diseases.

**Table 2:** Optimized and intensified process performance.

Accumulated VLPs	8.7E+13
Max. VLP concentration in a day (VLP/mL)	1.5E+10
VLP concentration in reactor (VLP/mL)	6.8E+10
Mean VLP specific productiviy (VLP·cell^−1^·day^−1^)	3.0E+03
Volumetric productivity / Reactor (VLP·L^−1^·day^−1^)	7.1E+12
Volumetric productivity / Spent medium (VLP·L^−1^·day^−1^)	2.7E+12
Total volume of spent media (L)	3.8

## Data Availability Statement

The original contributions presented in the study are included in the article/supplementary materials, further inquiries can be directed to the corresponding authors.

## Author Contributions

JL-G designed and performed the experiments, analyzed and interpreted the data and wrote the manuscript. LC and FG supervised the experiments and edited the manuscript. All authors contributed to the article and approved the submitted version.

## Conflict of Interest

The authors declare that the research was conducted in the absence of any commercial or financial relationships that could be construed as a potential conflict of interest.

## Abbreviations

ATF: Alternating tangential flow
CCD: Central composite design
CDE: Cell density effect
CSPR: Cell specific perfusion rate
DoE: Design of experiments
EGE: Extended gene expression
eGFP: enhanced green fluorescence protein
Gag::eGFP: translational fusion of HIV-Gag protein and eGFP
HFM: Hollow fiber module
Hpt: hours post transfection
PBS: Phosphate-buffered saline
PEI: polyethyleneimine
PES: Polyethersulfone
RFU: Relative fluorescence units
RSM: Response surface methodology
FESEM: Field emission scanning electron microscopy
SF: Shake Flasks
SPR: Sequential Perfusion Recovery
STR: Stirred tank bioreactor
TGE: Transient gene expression
VLPs: Virus-like particles.
