# Corrigendum: An Alternative Perfusion Approach for the Intensification of Virus-Like Particle Production in HEK293 Cultures

**DOI:** 10.3389/fbioe.2021.708773

**Published:** 2021-10-08

**Authors:** Jesús Lavado-García, Laura Cervera, Francesc Gòdia

**Affiliations:** Grup d’Enginyeria Cellular i Bioprocés, Escola d’Enginyeria, Universitat Autònoma de Barcelona, Barcelona, Spain

**Keywords:** bioreactor, perfusion, ATF, design of experiments, VLP, HFM

In the original article, there was a mistake in Figure 2 and its legend as published. The values 51.5 and 100 that appear in the figure and in section 2K of the legend should be 515 and 1000. The correct Figure 2 and its legend appears below.

**FIGURE 2:**
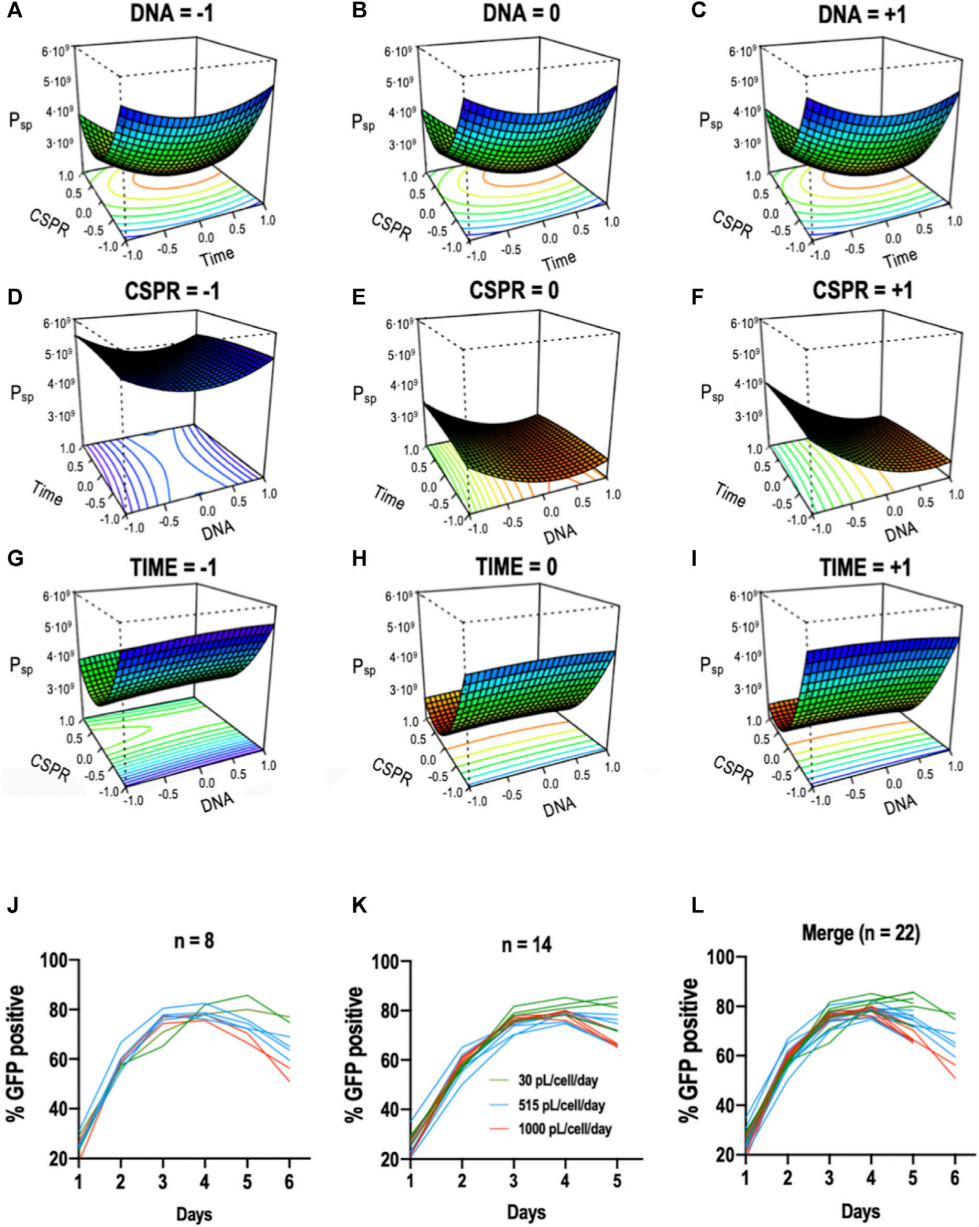
Response surface graphs based on Box-Behnken experimental results. Maximum VLP specific production in cell culture supernatants as a function of **(** **A** -**C** **)** CSPR vs. Time; **(** **D** -**F** **)** time vs. DNA; and **(** **G** -**I** **)** CSPR vs. DNA. The graphs were constructed by depicting two variables at a time and maintaining the third one at a fixed level. +1, 0, and −1 correspond to 0.5, 1.25, and 2 mg/ml for the DNA concentration; 30, 515, and 1000 pL/cell/day for the CSPR and 24, 48, and 72 hpt for the time of retransfection. **(** **J** -**L** **)** represent the changes of the percentage of transfection measured in percentage of GFP positive cells along the studied time course for conditions reaching the sixth day of the process **(** **J** **)** and conditions reaching the fifth **(** **K** **)**. **(** **L** **)** Shows the merged plot.

In the original article, there was also a mistake in Table 1 as published. The values 51.5 and 100 that appear in Table 1 for CSPR limits should be 515 and 1000. The corrected Table 1 appears below.

**TABLE 1:** Box-Behnken design, results and ANOVA analyses for optimization of the extended gene expression (EGE) protocol for VLP production.

		−1	0	1
Retransfection time (hpt)		24	48	72
CSPR (pL/cell/day)		30	515	1000
DNA (mg/mL)^a^		0.5	1.25	2
**Experimental run**	**Retransfection time**	**CSPR**	**DNA/mL**	**P**
1	1	0	−1	1.83E +09
2	1	0	−1	2.20E +09
3	−1	0	−1	2.68E + 09
4	−1	0	−1	3.29E + 09
5	1	0	1	1.85E + 09
6	1	0	1	1.92E + 09
7	0	−1	−1	5.06E + 09
8	0	−1	−1	4.33E + 09
9	0	1	−1	2.32E + 09
10	0	1	−1	2.36E + 09
11	0	1	1	2.10E + 09
12	0	1	1	2.47E + 09
13	−1	1	0	4.64E + 09
14	−1	1	0	2.91E + 09
15	1	−1	0	5.21E + 09
16	1	−1	0	5.56E + 09
17	0	0	0	2.07E + 09
18	0	0	0	1.92E + 09
19	0	0	0	1.72E + 09
20	0	0	0	1.89E + 09
21	0	−1	1	5.19E + 09
22	0	−1	1	4.10E + 09
23	1	1	0	2.25E + 09
24	1	1	0	1.94E + 09
25	−1	0	1	2.40E + 09
26	−1	0	1	4.07E + 09
27	−1	−1	0	5.65E + 09
28	−1	−1	0	5.19E +09
29	0	0	0	2.12E + 09
30	0	0	0	2.38E + 09
**Model**	**Multiple R^2^**	***P* value^b^**	**Lack of fit^c^**	—
—	0.9126	1.06E−08	0.9316	—
**Parameters**	**Coefficient**	**—**	***t***	***P* Value^b^**
Constant	0.02E + 09	—	10.225	<0.0001
(Time)	−5.04E + 08	—	−4.1761	0.0005
(CSPR)	−1.21 + 09	—	−9.9874	<0.0001
(DNA)	1,88E + 06	—	0.0155	0.988
(Time)-(CSPR)	−4.11E + 08	—	−2.4077	0.026
(Time)-(DNA)	−9.50E + 07	—	−0.5562	0.584
(CSPR)-(DNA)	−1.25E + 06	—	−-0.0073	0.994
(Time)^2^	5.95E + 08	—	3.3492	0.003
(CSPR)^2^	1.56E + 09	—	8.7562	<0.0001
(DNA)^2^	−8.21E + 07	—	−0.4617	0.649
**Optimal Values**	—	—	—	—
—	Time of Retransfection	CSPR	DNA	P_sp_
—	−1	−1	0.5977154	5.49E + 09
—	**At 24 hpt**	**30 pL/cell/day**	**1.7 μg/ml**	—

a DNA/PEI ratio was always maintained at (1:2)

b *p values* under 0.05 are considered statistically significant with 95 % confidence, and under 0.1, statistically significant with 90 %.

c *p values* associated to lack of fit test above 0.05 mean that the hypothesis arguing that the model is suitable cannot be rejected.

hpt: hours post transfection

In the original article, there was also an error in the text. The value of 3000 x*g* in the mentioned centrifugation should be 300 x*g*. A correction has been made to Section 2: **Experimental**, Sub-section 2.1: “*HEK293 mammalian cell line, culture conditions*,” Paragraph 3:

“For the pseudoperfusion experiments, the total culture volume was 20 ml and media replacement (MR) was carried out centrifuging the culture at 300 x*g* for 5 min every 12 h ensuring that the proportional volume of media was replaced depending on the condition. To maintain a MR rate of 2 reactor volume per day (RV/day), 20 ml were replaced every 12 h. For a rate of 1 RV/day, 10 ml were replaced every 12 h and for a rate of 0.5 RV/day, 5 ml were replaced every 12 h.”

Further, the value of 100 pL/cell/day should be 1000 pL/cell/day in accordance to the previous correction in Table 1 and Figure 2. A correction has been made to Section 3:** Results and Discussion**, Sub-section 3.2: “*Optimization of retransfection by Design of Experiment (DoE) method*,” Paragraph 1:

“As for the CSPR, the working range was set based on the previous study of the cell growth upon different media replacement rates. The upper limit was set at 1000 and the lower limit at 30 pL/cell/day.”

Finally, the value of 8.7·10^11^ VLP·L^-1^·day^-1^ should be 2.7·10^12^ VLP·L^-1^·day^-1^. This value is already correctly presented in **Table 2**. However, this typo was overlooked in the text when revising the manuscript. The value of 2.7·10^12^ VLP·L^-1^·day^-1^ is the one obtained in this work and 8.7·10^11^ is the one that it is being compared to.

A correction has been made to Section 3: **Results and Discussion**, Sub-section 3.4: *Intensification of the optimized protocol in bioreactor using ATF*, Paragraph 1:

“...the presented work achieved a reactor and media volumetric productivity of 7.1·10^12^ and 2.7·10^12^ VLP·L-1·day-1 respectively, improving 26.8% or 1.36 fold and 67.8% or 3.1 fold respectively.”

The authors apologize for these errors and state that this does not change the scientific conclusions of the article in any way. The original article has been updated.

